# Autologous Serum for the Treatment of Macular Holes: A Systematic Review and Meta-Analysis

**DOI:** 10.7759/cureus.66174

**Published:** 2024-08-05

**Authors:** Dillan Cunha Amaral, Tiago Nelson de Oliveira Rassi, Raiza Jacometti, Ariane Barros Mesquita Cunha, Lucas Cavinato Kwitko, Karina de Oliveira Caneca, Lucas de Sousa Tebicherane, Milton Ruiz Alves, Mário Luiz Ribeiro Monteiro, Mauricio Maia, Ricardo Noguera Louzada

**Affiliations:** 1 Ophthalmology, Federal University of Rio de Janeiro, Rio de Janeiro, BRA; 2 Ophthalmology, Foundation Bank of Eyes of Goiás, Goiânia, BRA; 3 Ophthalmology, University of São Paulo, São Paulo, BRA; 4 Dermatology, Federal University of Rio de Janeiro, Rio de Janeiro, BRA; 5 Ophthalmology, Pontifical Catholic University of Rio Grande do Sul, Porto Alegre, BRA; 6 College of Medicine, Federal University of Rio de Janeiro, Rio de Janeiro, BRA; 7 Ophthalmology, Federal University of São Paulo, São Paulo, BRA

**Keywords:** retina, autologous serum, macular hole, meta-analysis, systematic review

## Abstract

This systematic review and meta-analysis evaluated the efficacy and safety of autologous human serum as an adjuvant agent in pars plana vitrectomy (PPV) for macular holes (MH). Thus, a comprehensive search was conducted across PubMed, Web of Science, Embase, and the Cochrane Library databases up to August 20th, 2023. The inclusion criteria targeted randomized clinical trials (RCTs) or non-RCTs that compared the use of autologous serum in vitrectomy for MH with the same procedure without the serum. The outcomes were MH closure rates and postoperative complications such as retinal detachment and cataracts. Odds ratios (OR) and mean differences (MDs) were calculated using a random-effects model. Review Manager 5.3 (The Cochrane Collaboration, Oxford, UK) was used for statistical analysis. Four studies, comprising two RCTs and two non-randomized cohort studies with 373 eyes of 372 patients, were included. The pooled analysis showed no significant difference in MH closure rates (OR 1.28; 95% confidence interval (CI): 0.48 to 3.43; P=0.62) and no difference concerning the incidence of adverse events (OR 0.97; 95% CI: 0.30-3.09; P=0.96). Leave-one-out sensitivity analysis excluding the study by Lauritzen et al. revealed a significant difference in anatomical closure, favoring the serum arm, and demonstrated a reduction in the level of heterogeneity. Our meta-analysis demonstrated no difference between groups in the pooled analysis of all studies. However, considering the quality assessment of one of the included studies, and observing the divergent result in sensitivity analysis following its exclusion, there are indications that might suggest the superiority of the serum in terms of the analyzed endpoints. This finding highlights the existing research gaps and the imperative need for additional high-quality randomized trials to further investigate this treatment.

## Introduction and background

In 1869, the first description of a macular hole (MH) emerged, attributing its genesis to a traumatic incident [1]. MH is characterized by a vertical defect in the neurosensory retinal anatomy, primarily within the foveal region, extending from the internal limiting membrane (ILM) to the retinal pigment epithelium (RPE) [2-4]. It profoundly impacts central vision and induces metamorphopsia. Most MHs are primary and idiopathic, while secondary MHs can be associated with factors such as high myopia, trauma, proliferative diabetic retinopathy, and various retinal pathologies [5].

The gold standard treatment for MH is surgical intervention involving pars plana vitrectomy (PPV) with peeling of the ILM, followed by gas tamponade. This technique presents with high closure rates and satisfactory improvement on best corrected visual acuity (BCVA) [6,7]. However, challenging cases, such as large MHs, myopic MHs, or MHs associated with retinal detachments (RDs), are linked to poorer outcomes [1], which has led to the advent of alternative treatment methods, such as inverted ILM flap, lens capsular flap transplantation [8], tapping of MH edges [9], autologous platelet concentrates [10] and autologous human serum [11].

Growth factors like epidermal growth factor (EGF), vascular endothelial growth factor (VEGF), and platelet-derived growth factor (PDGF) in autologous serum have been shown to promote cell proliferation and wound healing in vitro, with literature supporting its efficacy on human retinal pigment epithelial cell migration and proliferation [12,13]. This potential benefit for MH surgery using the serum was shown by Liggett et al., reporting a 100% resolution of the surrounding subretinal fluid and flattening of the hole [14]. Other authors confirmed these findings with high success in closure rates and better visual outcomes [15]. Despite the potential benefits of autologous serum and the existing literature on its potential advantages, further research is necessary to explore its applicability in treating MH [11,16].

Therefore, in order to confirm the potential benefits of using autologous serum, we conducted this systematic review and meta-analysis of all available studies to assess autologous human serum's comparative efficacy and safety in PPV for MH.

## Review

Methods

Protocol and Registration

The protocol for this meta-analysis followed the Cochrane Handbook for Systematic Reviews of Interventions and Preferred Reporting Items for Systematic Reviews and Meta-Analysis (PRISMA) method [17]. This study was registered in the International Prospective Register of Systematic Reviews (PROSPERO) with ID CRD42023475614.

Search Strategy and Data Extraction

We systematically searched PubMed, Web of Science, Embase, and the Cochrane Library databases from inception to August 20th, 2023, with no language restrictions. The search strategy was as follows: (("Retinal Perforation" OR "Holes, Retinal" OR "Macular Hole" OR "Macular Holes" OR "Retinal Break" OR "Retinal Breaks" OR "Retinal Dialyses" OR "Retinal Hole" OR "Retinal Holes" OR "Retinal Perforation" OR "Retinal Tear" OR "Retinal Tears") AND ("Autologous Serum" OR “Blood Serum” OR Serums)) was used for the search. The references from all included studies, previous systematic reviews, and meta-analyses were also searched manually for any additional eligible studies. The eligibility assessment was performed independently by two reviewers (A.C. and D.A.). Duplicates were identified and removed. The titles and abstracts were first screened for inclusion, and full manuscripts were reviewed and assessed if necessary. Discrepancies were resolved and discussed by consulting the corresponding author (R.L.).

Inclusion and Exclusion Criteria

The inclusion criteria for this research were (1) randomized clinical trials (RCTs) and observational studies; (2) evaluating patients with MH who underwent PPV using autologous serum as an adjuvant agent; (3) comparing to PPV without autologous serum; (4) reporting at least one or more clinical outcomes of interest. The exclusion criteria were as follows: (1) non-comparative single-arm studies, case reports, or series and animal studies; (2) abstracts, editorials, letters, and conference proceedings without efficient data; and (3) interface pathologies that do not encompass a full-thickness MH, such as lamellar holes, pseudo holes.

Outcomes

The outcomes of interest were MH closure, visual acuity, general complications, and specific complications, including postoperative RD, postoperative cataract, and postoperative endophthalmitis. The incidence of any complications reported was evaluated. RD, pigment change in the macula, epiretinal membrane, keratoconjunctivitis, cataract, elevated intraocular pressure, and endophthalmitis were reported.

Subgroup Analysis and Leave-One-Out Analysis

Subgroup analysis was conducted based on the design of the article (RCT or observational) to identify if heterogeneity of results would arise from different design settings. A leave-out-one sensitivity analysis was also conducted to determine each study's effect on the estimated pooled analysis by excluding each study step-by-step.

Risk of Bias Assessment

The risk of bias in RCTs was assessed using version 2 of the Cochrane risk of bias (ROB-2) assessment tool. Each study is labeled as “low risk”, “some concerns”, or “high risk” of bias according to each domain evaluation [18]. Non-randomized studies were assessed with the risk of bias in non-randomized studies - of interventions tool (ROBINS-I) [19]. Two independent authors completed the risk of bias assessment (D.A. and K.C.). Disagreements were resolved through a consensus after discussing reasons for the discrepancy.

Statistical Analysis

This systematic review and meta-analysis were performed in accordance with the Cochrane Collaboration and the PRISMA statement guidelines. Odds ratios (OR) with 95% confidence intervals (CIs) were used to compare treatment effects for categorical endpoints. Continuous outcomes were compared with mean differences (MD). Heterogeneity across studies was evaluated using Cochran’s Q test, I^2^ test, and τ^2^ test. An I^2^ value greater than 50% indicated high statistical heterogeneity, for which a random-effects model was used. Review Manager 5.3 (The Cochrane Collaboration, Oxford, UK) was used for statistical analysis.

Results

Study Selection and Characteristics

**Figure 1:**
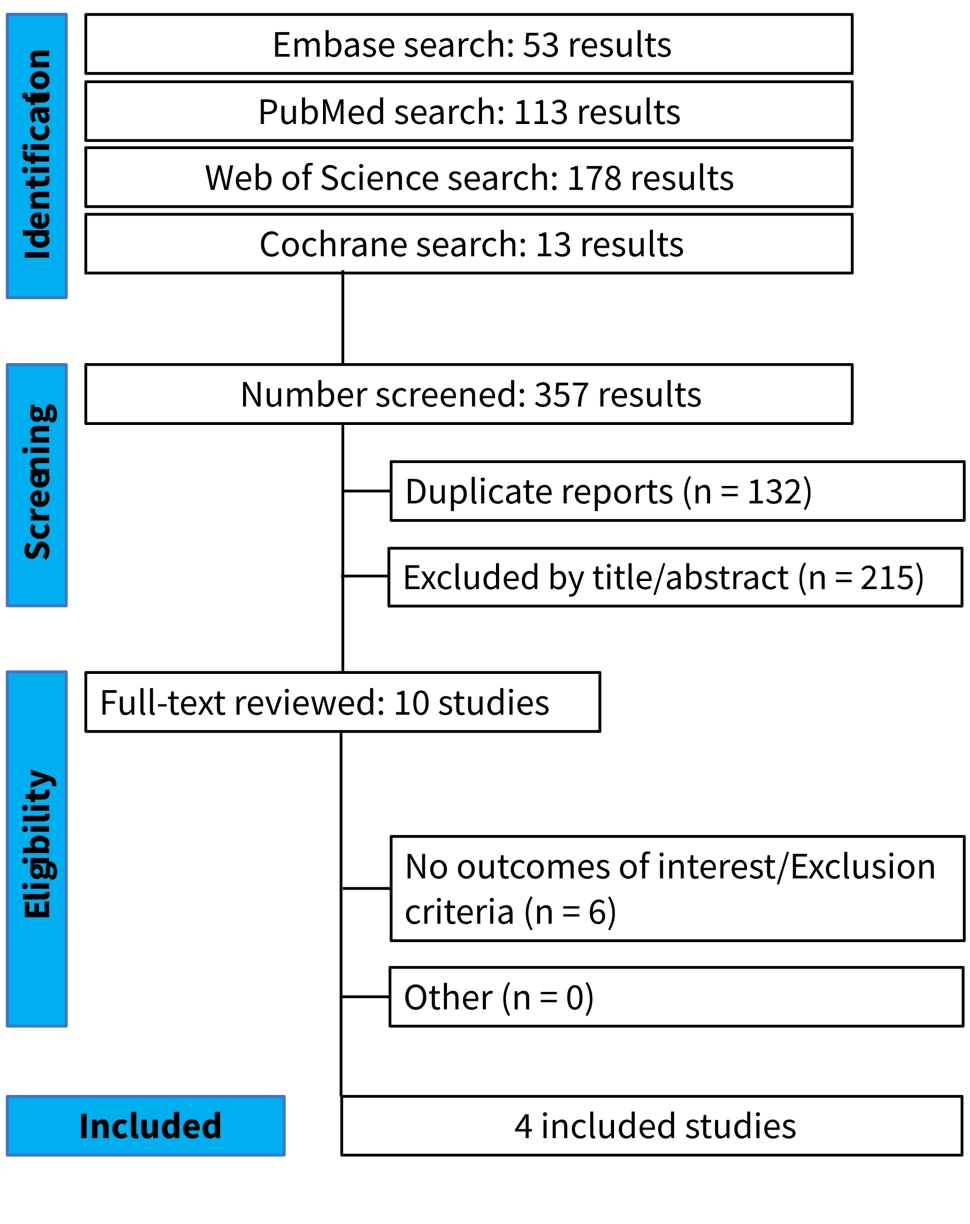
PRISMA Flow Diagram of Study Screening and Selection PRISMA: Preferred Reporting Items for Systematic Reviews and Meta-Analysis

Initial search yielded a total of 359 articles, including 113 in PubMed (MedLine), 53 in Embase (Elsevier), 178 in Web of Science, and 13 in Cochrane databases (Figure 1).

Of these, 132 duplicates were removed. After the removal of duplicate records and ineligible studies, 10 remained and were fully reviewed based on inclusion criteria. Next, six articles were excluded as per our exclusion criteria. Finally, four studies were included in this review, two RCTs [20,21] and two non-randomized cohorts [11,16], comprising a total of 372 patients. The mean age was 66.63 ± 7.55 years in the serum arm and 66.67 ± 7.94 in the control arm. A total of 373 eyes were analyzed, 217 in the serum arm and 159 in the control arm. The follow-up period ranged from six months to two years. Study characteristics are summarized in Table 1.

**Table 1:** Baseline Characteristics OB: observational; NA: not applicable; w: weeks; Preo: before surgery; RCT: randomized clinical trial; MH: macular hole

Author (Year)	Study type	Eyes (serum: control)	Country	Mean age (serum)	Mean age (control)	Mean vs Preop (serum)	Mean vs Preop (control)	Face down duration	Base diameter of the hole (µM) (serum)	Base diameter of the hole (µM) (control)	MH stage (1/2/3/4) (serum)	MH stage (1/2/3/4) (control)	Follow-up (months)
Banker et al., 1999 [16]	OB	106:58	EUA	67.2 ± 8.6	67.2 ± 8.6	0.87 ± NA	0.77 ± NA	2w	447.5 ± 152.6	541.1 ± 159.2	NA	NA	6
Erza and Gregor​​​​, 2004 [21]	RCT	65:59	England	66.69 ± 5.97	67.15 ± 7.94	0.68 ± 0.20	0.67 ± 0.22	2w	370.64 ± 141.13	399.67 ± 147.49	(0/32/27/6)	(0/18/34/7)	24
Kung and Wu, 2013 [11]	OB	19:19	China	63.89 ± 6.62	60.79 ± 5.21	1.08 ± 0.21	1.01 ± 0.32	2w	NA	NA	(0/3/5/11)	(0/9/4/6)	7
Lauritzen et al., 2003 [20]	RCT	24:23	EUA	66.13 ± NA	69.70 ± NA	1.05 ± NA	0.98 ± NA	2w	321 ± NA	344 ± NA	NA	NA	7

Pooled Analysis of All Studies

**Figure 2:**
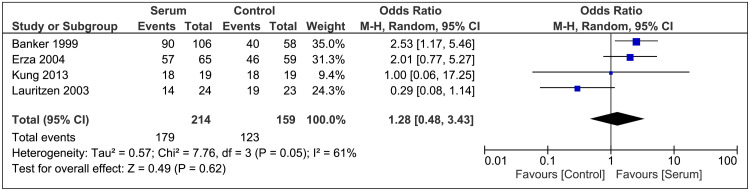
Anatomical Closure Forest Plot References [16,21,11,20]

MH closure: All the included studies reported the incidence of MH closure after surgery. The pooled results revealed no significant difference between patients in the autologous serum group and control group (OR 1.28; 95% CI 0.48 to 3.43; P=0.62; I²=61%; Figure 2).

Complications

**Figure 3:**
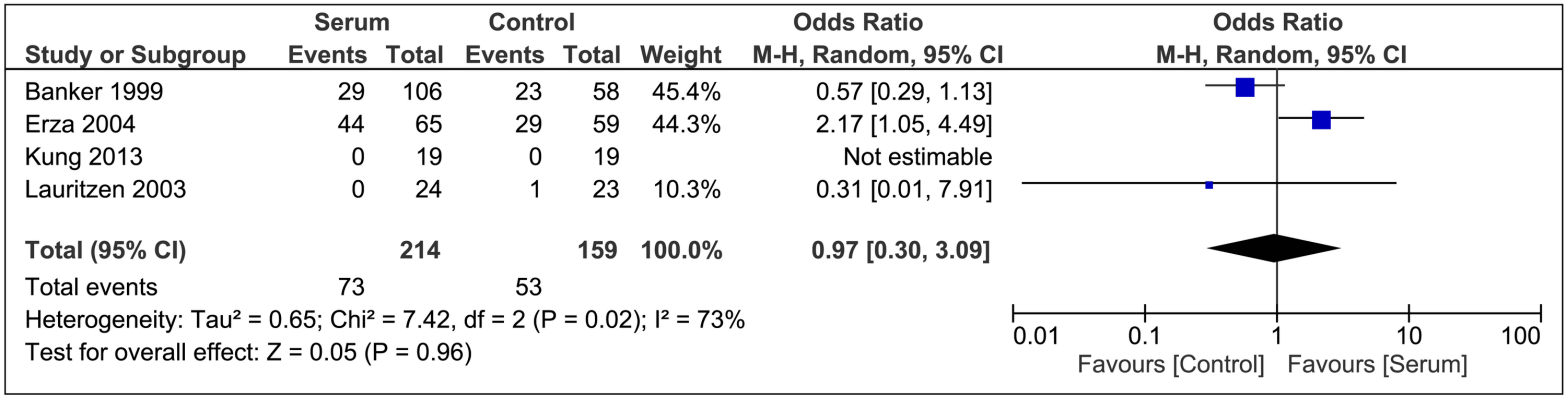
Complications Forest Plot References [16,21,11,20]

After combining the total events of complications, 126 events were reported. The occurrence of complications wasn’t significantly different between the two groups (OR 0.97; 95% CI 0.30-3.09; P=0.96; I²=73%; Figure 3).

Incidence of Postoperative RD

**Figure 4:**
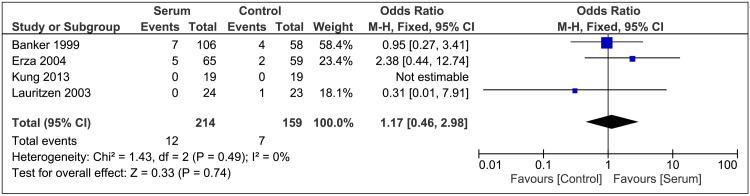
Retinal Detachment Forest Plot References [16,21,11,20]

For postoperative RD, all included studies stressed the postoperative incidence, with OR 1.17; 95% CI 0.46-2.98; P=0.74; I²=0% (Figure 4). The results showed no significant difference between the two groups.

Incidence of Postoperative Cataract

**Figure 5:**
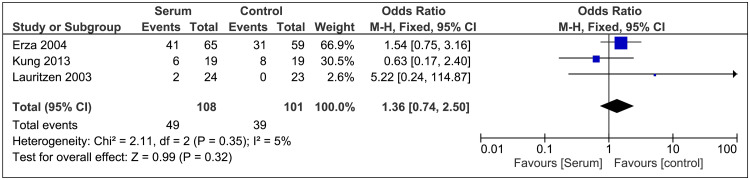
Cataract Forest Plot References [21,11,20]

The incidence of postoperative cataracts was reported in three studies [11,20,21]. The pooled analysis showed that OR 1.36; 95% CI 0.74-2.50; P=0.32; I²=5% (Figure 5) for the comparison between the autologous serum group and the control group. The results showed no significant difference between the two groups.

Incidence of Postoperative Endophthalmitis

Three studies [11,16,20] reported the incidence of postoperative endophthalmitis, and the rates were all zero in both the autologous serum and control groups.

Leave-One-Out Sensitivity Analysis

Performing the leave-one-out analyses in MH closure, when the study conducted by Lauritzen et al. was excluded, anatomical closure exhibited statistical significance, suggesting a more favorable outcome for the serum arm. There was a reduction in heterogeneity (I^2^=61% to I^2^=0%) after excluding this study [20]. In the complications outcome, the leave-one-out analysis did not reveal any significant deviation from the primary findings (Table 2).

**Table 2:** Leave-One-Out Analysis OR: odds ratio; CI: confidence interval; MH: macular hole

Study omitted	Pooled analysis MH closure	Pooled analysis complications
Banker et al., 1999 [16]	OR 0.87; CI 0.21 to 3.56; p=0.84; I²=61%	OR 1.58; CI 0.39 to 6.49; p=0.52; I²=25%
Erza and Gregor, 2004 [21]	OR 0.96; CI 0.19 to 4.87; p=0.96; I²=73%	OR 0.56; CI 0.29 to 1.08; p=0.09; I²=0%
Kung and Wu, 2013 [11]	OR 1.29; CI 0.41 to 4.00; p=0.66; I²=74%	OR 0.97; CI 0.30 to 3.09; p=0.96; I²=73%
Lauritzen et al., 2003 [20]	OR 2.23; CI 1.24 to 4.02; p=0.007; I²=0%	OR 1.11; CI 0.30 to 4.08; p=0.88; I²=85%

Subgroup Analysis

A subgroup analysis was conducted based on the type of study. In RCTs, the pooled results indicated no significant difference between patients treated with autologous serum and controls (OR 0.82; 95% CI 0.12 to 5.37; P=0.83; I²=81%). Conversely, in observational studies, the pooled results also showed no significant difference between patients treated with autologous serum and controls (OR 2.38; 95% CI 1.13 to 4.99; P=0.02; I²=0%).

Risk of Bias Assessment

**Figure 6:**
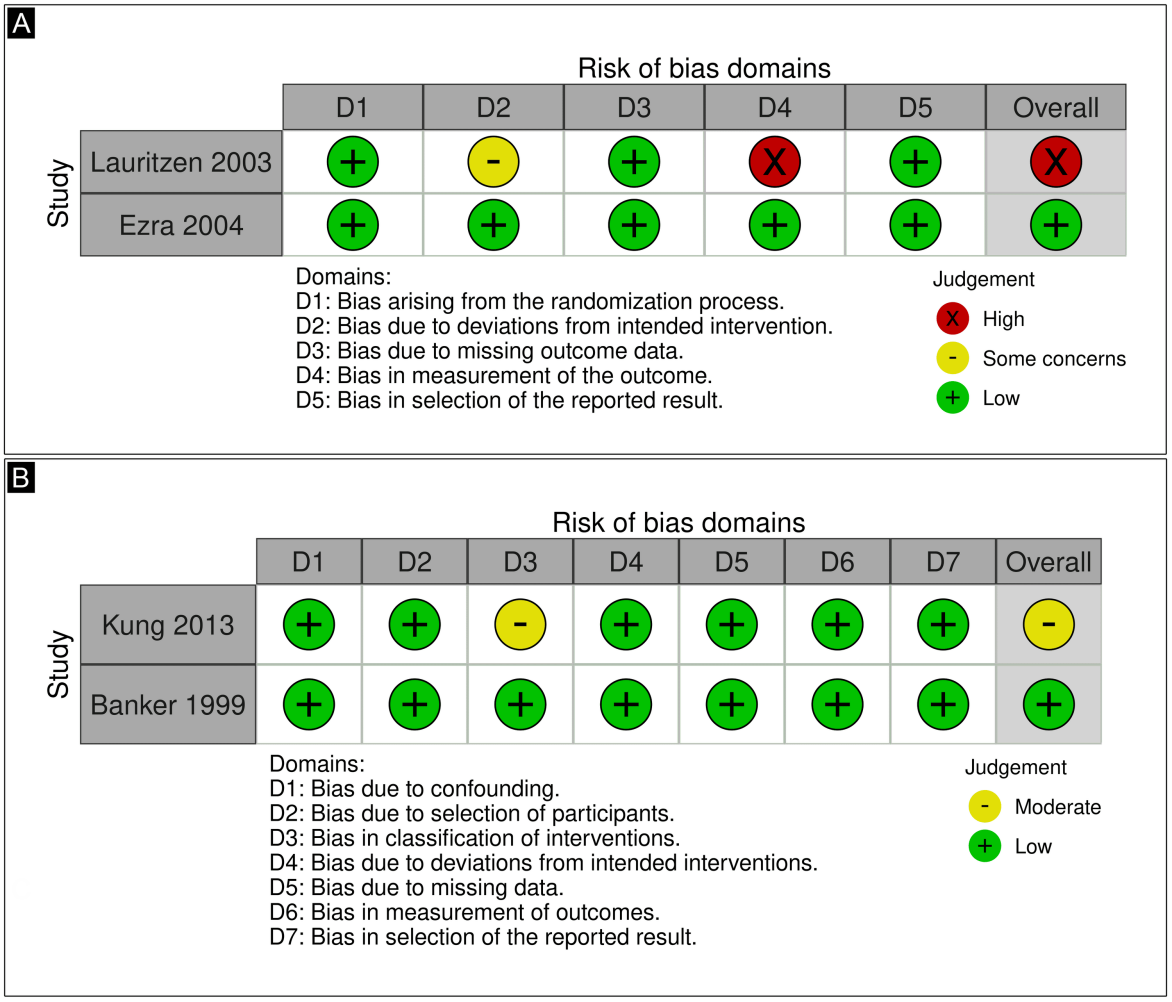
(A) Risk of bias assessment of RCTs using the ROB-2 tool; (B) Risk of bias assessment of non-RCTs using the ROBINS-I. RCTs: randomized clinical trials; ROB-2: version 2 of the Cochrane Risk of Bias; ROBINS-I: risk of bias in non-randomized studies - of interventions tool References [20,21,11,16]

The risk of bias appraisal for individual studies is reported in Figure 6. One study was deemed as having some concerns about bias in the classification of interventions [11]. One study was deemed as having a high risk for bias in the measurement of outcomes [20]. The other two studies were classified as having a low risk of bias [16,21]. 

Discussion

To our knowledge, this meta-analysis is the first to compare the use of autologous serum with conventional treatment of MH. Previous meta-analyses related to autologous serum have mainly focused on acupoint injection [22], chronic urticaria [23], and dry eye disease [24]. We found in our pooled analyses that the serum group did not show statistical differences when compared with the control group regarding closure rates and complication rates.

In the sensitivity analysis, human serum showed more efficacy in closure rates when compared to conventional treatment when excluding the study by Lauritzen et al. The study in question showed a minimal variance in MH closure rates between groups and had a great influence on the high heterogeneity level according to the leave-one-out sensitivity analysis. In a further look into that study, there were some methodological inconsistencies that could have led to biases. Even though patients were randomized, some individuals in the serum group used sulphur hexafluoride (SF6) gas tamponade while all patients in the control group used perfluoropropane (C3F8) gas tamponade. Among the three eyes from the serum group treated with SF6, two had an anatomical failure with non-closure of the MH. The efficacy of SF6 gas tamponade is highly debatable, with some studies showing inferior efficacy compared to C3F8 [25-27]. Considering the quality assessment of the study mentioned and the divergent result in sensitivity analysis following its exclusion, there are indications that might suggest the superiority of the serum in terms of the analyzed endpoints. These findings highlight the existing research gaps and the imperative need for additional high-quality randomized trials to further investigate this treatment.

Furthermore, there is evidence in the literature suggesting that autologous serum presents advantages, particularly in cases featuring large MHs. This is supported by Banker et al., where larger MH diameters (447.5±152.6 µm) were associated with better outcomes in achieving anatomical closure [16]. Due to the lack of data, we were unable to test the efficacy of serum in larger and chronic MHs. Better outcomes in these cases are expected due to favorable results observed in similar therapies, such as autologous platelets, in the treatment of large MHs [1,28,29].

Regarding adverse events, previous literature shows a variety of results. Ezra's study reported lower complication rates in the control group compared to the serum group, while Banker et al.'s study found a lower rate in the intervention group [16,21]. This inconsistency was also noted in cases of retinal detachment and postoperative cataracts, with the frequency of these complications varying between the two studies. Despite this variability and contrasting results, our pooled analysis showed no significant difference between both groups, which helps clarify this issue and suggests the use of serum as a safe treatment.

Our meta-analysis is subject to several limitations. Firstly, the scarcity of RCTs is a potential source of bias. Discrepancies in post-surgery evaluation timings (from six months to two years) and differing definitions of MH stages across studies hindered the comparison of long-term effects. Furthermore, variations in vitrectomy gauge, tamponade materials, MH type, and the use of optical coherence tomography assessments among studies exacerbated this heterogeneity [30]. We were unable to test the efficacy of serum in larger and chronic MHs due to a lack of data. Some studies suggest the use of serum may be beneficial in larger MHs, but it couldn’t be analyzed in our study [21]. To address these issues, we performed a series of sensitivity tests and subgroup analyses.

## Conclusions

To the best of our knowledge, this is the first meta-analysis on this theme. Although the pooled analysis of all studies showed no significant difference between groups, the quality assessment of one included study and the divergent results observed in the sensitivity analysis following its exclusion suggest that the serum may be superior to the control in anatomical outcomes without difference in adverse events. Furthermore, large-sample RCTs are necessary to support these meta-analysis results.
